# Municipalities and the Voluntary Principle

**Published:** 1917-04-07

**Authors:** 


### MUNICIPALITIES AND THE VOLUNTARY PRINCIPLE.

The Hospital has long advocated the closer co-operation of municipal authorities with the work of hospitals, on which recent legislation makes local bodies increasingly rely for the treatment of disease, especially among women and children. We have advocated an extension of an existing principle as just in itself, as logical in itself, and the best way of maintaining the voluntary principle under the new burdens which the State is asking hospitals to shoulder. It used to be thought that there was no half-way house between voluntary charity and municipalisation; but we have known too well the vitality of the voluntary principle to believe for one moment that it could be impaled on either horn of this logical, but in fact unreal, dilemma. Liverpool is one of the cities which have set an example in this respect, and therefore we attach importance to a recent speech in which the Lord Mayor emphasised the value of the co-operation for which we have pressed so long.

In presiding at a recent meeting of the Liverpool Maternity Hospital and Ladies' Charity, the Lord Mayor referred gratefully to the work this body was doing in training midwives at the Babies' Welcome and at the Rest Home. It deserved every encouragement, and already received a small grant of £70 from the Liverpool Education Committee. He hoped that the public would demand a broad-based scheme whereby the Liverpool Corporation could assist these excellent institutions, in return perhaps for a certain amount of representation. He could not speak for the Council in the matter of finance, but he was most strongly impressed with the need for some closer links such as he had suggested. Other speakers supported the contention, and we have reason to believe that he voiced a public-spirited movement in his words.

It is, therefore, well again to consider the relation of municipalities to hospitals, and to point out that some such scheme as was outlined by the Lord Mayor is not the thin end of the wedge, inserted in the foundations of the voluntary system. Conservative hospitals still fear it, but we would ask them whether the voluntary principle has permanently suffered by the creation and influence of the huge working-class funds of the Midland and Northern cities. It would be idle to deny that the representation which accompanies these funds has created difficulties; it has required, indeed, clear heads, strong hands, and almost a special technique of management to guide the movement into proper channels. But, in spite of all this, the Midland and Northern hospitals are not "in the pockets of the trade unions," to which it was once feared their dependence on workshop collections would reduce them. Leicester Royal Infirmary is a shining example to the contrary. There the workmen have been permeated by the voluntary principle. If this be so in the extreme case of labour organisations, somewhat flushed at their new influence, the solution will be much easier when the aid of municipalities is invoked, for municipalities as a rule have their fill of authority and management.

In short, the grant in aid given by a municipality to a voluntary hospital for the work which it relies on that institution to perform is the solution. It is a legitimate form of co-operation, which enables new spheres of work to be undertaken, and thereby is rather an extension of the voluntary hospital's usefulness than an infringement of its rightly cherished character. It is because the voluntary principle has the vitality to meet these changes and adapt its form to them that it is assured of elasticity and permanence, and we welcome the far-seeing speech of the Lord Mayor of Liverpool as a happy example of municipal statesmanship, which voluntary hospital managers would do well to take to heart.

